# Identifying high or low risk of mother to child transmission of HIV: How Harare City, Zimbabwe is doing?

**DOI:** 10.1371/journal.pone.0212848

**Published:** 2019-03-13

**Authors:** Brian Komtenza, Srinath Satyanarayana, Kudakwashe C. Takarinda, Solomon H. Mukungunugwa, Owen Mugurungi, Prosper Chonzi, Ngwarai Sithole, Talent Bvochora, Angela Mushavi

**Affiliations:** 1 AIDS and TB Unit, Ministry of Health and Child Care, Government of Zimbabwe, Harare, Zimbabwe; 2 Center for Operational Research, International Union Against Tuberculosis and Lung Disease, Paris, France; 3 City of Harare Health Services Department, Harare, Zimbabwe; University of North Carolina at Chapel Hill, UNITED STATES

## Abstract

**Background:**

Despite high antiretroviral (ARV) treatment coverage among pregnant women for prevention of mother-to-child transmission (PMTCT) of Human Immunodeficiency Virus (HIV) in Zimbabwe, the MTCT rate is still high. Therefore in 2016, the country adopted World Health Organization recommendations of stratifying pregnant women into “High” or”Low” MTCT risk for subsequent provision of HIV exposed infant (HEI) with appropriate follow-up care according to risk status.

**Objective:**

The study sought to ascertain, among pregnant women who delivered in clinics of Harare in August 2017: the extent to which high risk MTCT pregnancies were identified at time of delivery; and whether their newborns were initiated on appropriate ARV prophylaxis, cotrimoxazole prophylaxis, subjected to early HIV diagnostic testing and initiated on ARV treatment.

**Methods:**

Cross-sectional study using review of records of routinely collected program data.

**Results:**

Of the 1,786 pregnant women who delivered in the selected clinics, HIV status at the time of delivery was known for 1,756 (98%) of whom 197 (11%) were HIV seropositive. Only 19 (10%) could be classified as “high risk” for MTCT and the remaining 90% lacked adequate information to classify them into high or low risk for MTCT due to missing data. Of the 197 live births, only two (1%) infants had a nucleic-acid test (NAT) at birth and 32 (16%) infants had NAT at 6 weeks. Of all 197 infants, 183 (93%) were initiated on single ARV prophylaxis (Nevirapine), 15 (7%) infants’ ARV prophylaxis status was not documented and one infant got dual ARV prophylaxis (Nevirapine+Zidovudine).

**Conclusion:**

There was paucity of data requisite for MTCT risk stratification due to poor recording of data; "high risk" women were missed in the few circumstances where sufficient data were available. Thus "high risk" HEI are deprived of dual ARV prophylaxis and priority HIV NAT at birth and onwards which they require for PMTCT. Health workers need urgent training, mentorship and supportive supervision to master data management and perform MTCT risk stratification satisfactorily.

## Introduction

Most of the 1.8 million human immunodeficiency virus (HIV) infected children are in sub-Saharan Africa [1], where the majority (~90%) acquired infection through mother-to-child transmission (MTCT) either during pregnancy, at the time of delivery or during breastfeeding.[2][3] As one of the strategies to end the global AIDS epidemic by 2030, it is necessary to eliminate new HIV infections in children.[4]

With comprehensive prevention of MTCT (PMTCT) interventions, MTCT rates which range from 15–45% can fall to ≤5%.[2] However, due to challenges in the optimal implementation of PMTCT interventions, globally in 2016, approximately 160,000 children were newly infected with HIV, mainly in Sub-Saharan Africa [5]

World Health Organization (WHO) released new PMTCT guidelines in 2015, which recommends identifying pregnant and lactating women who are at “high-risk” of MTCT. HIV-infected pregnant women who fulfill one of the four criteria are considered as ‘high-risk’: i) maternal viral load (VL) >1,000 copies/mL at ≥32 weeks of gestation; ii) are ARV-naive; iii) are on ARV treatment for <8 weeks before delivery; iv) or have incident HIV infection (sero-conversion) during pregnancy or breastfeeding. [6] Those without any of these four criteria are considered as ‘low-risk’ of MTCT. Risk stratification helps in identifying HIV-exposed infants (HEI) born of high-risk pregnancy mothers who need: i) intensive antiretroviral (ARV) prophylaxis; ii) early infant diagnosis (EID) at birth; and, iii) early initiation of ARV treatment. [6][7] The intensive ARV prophylaxis is a dual ARV prophylactic drug regimen, Nevirapine (NVP) and Zidovudine (AZT) instead of mono ARV prophylaxis with NVP. This is in line with WHO recommendation following evidence from the HPTN 040 and HPTN 046 randomized clinical trials which showed significant reduction of MTCT of HIV with dual and triple ARV prophylaxis compared to mono ARV prophylaxis and significant reduction in MTCT with extended ARV drug prophylaxis versus 6 week ARV prophylaxis respectively among other evidence. [6][8][9]

Zimbabwe, in Southern Africa, is among the five countries worst affected by HIV. Since Zimbabwe’s adoption in 2012 of WHO recommendations for ART scale-up among pregnant women, [10] ART coverage rapidly increased to 84% among estimated population of pregnant women living with HIV by December 2015. [1] Despite this, in 2016 Zimbabwe’s MTCT case rate was ~621 new HIV infections per 100,000 live births; well above the elimination of MTCT (EMTCT) targets of ≤50 per 100,000 live births.[5,11] Zimbabwe adopted the new 2015 WHO PMTCT guidelines in the year 2016 and is aiming to achieve EMTCT.

In efforts to reach EMTCT targets, there has been strong demand from the EMTCT program to ascertain the implementation status of risk differentiation of pregnancies in HIV positive women, and the management of their HIV exposed babies. Therefore, this study was undertaken in delivery clinics of Harare (the capital city of Zimbabwe) to ascertain: a) the extent to which high risk MTCT pregnancies were identified at time of delivery; and b) whether the newborn babies were initiated on appropriate ARV prophylaxis, cotrimoxazole prophylaxis and subjected to early HIV diagnostic testing and initiated on ART.

## Methods

### Study design

This was a cross-sectional study involving secondary analysis of routinely collected program data.

### Setting

Harare has a population of approximately 1.6 million people. Aside from two central hospitals, 43 health clinics under Harare City Health Department (of which two are infectious disease hospitals whilst the rest are polyclinics or satellite clinics) offer public health care services including PMTCT. The EMTCT program started in the country in 1999.

According to the 2016 EMTCT guidelines all pregnant women who enroll for ANC receive HIV testing. Women who test negative on enrolment are retested for HIV throughout the pregnancy and breastfeeding period. [12] The women who test positive are initiated on lifelong ARV (Tenofovir+ Lamivudine+ Efavirenz_600_) preferably on the same day. Once enrolled in the EMTCT program, women who are already on ARV treatment receive a VL test if it was not done in previous 6 months. If the VL is suppressed, it is repeated again in the third trimester (> 32 weeks gestational age). In an ARV naïve woman, VL test is done after three months on ARV treatment and repeated in the third trimester. [12]

HIV-positive pregnant women are assessed for the risk of MTCT and classified as either ‘high’ or ‘low’ risk. Services for infants born to ‘high risk’ women are as follows: early infant diagnosis (EID) using nnucleic acid testing (NAT) at birth; if negative, NAT is repeated at 6 weeks. Infants testing positive to NAT are initiated on ART upon diagnosis. Those negative at 6 weeks receive a rapid HIV test at 9 and 18 months and if either are positive, they have a confirmatory NAT. Infants born to “low risk” women receive routine HIV NAT at 6 weeks and thereafter the HIV testing and ART protocol are similar to the high risk.[12]

All infants born to HIV-positive mothers are offered antiretroviral prophylaxis from birth according to risk classification and infant feeding method. Infants born to ‘high risk’ pregnancies who are breastfed receive nevirapine (NVP) plus zidovudine (AZT) for 12 weeks whilst their non-breastfed counterparts will receive the same combination for 6 weeks. Infants born to ‘Low risk’ pregnancies receive NVP from birth to 6 weeks regardless of infant feeding method. All infants regardless of risk category receive Cotrimoxazole prophylaxis from 6 weeks. [12]

### Patient population

The study included all HIV positive pregnant women (n = 1,786) who delivered in August 2017 at the 12 delivery clinics under the Harare City health department and were documented in the delivery registers.

### Sources of data, data variables and data collection

Data were collected from delivery registers, antenatal (ANC) registers, HEI follow-up registers and ART registers. For each HIV-positive pregnant woman who delivered during the study period, the following data meant to be collected: registration number (as per delivery registers), name, date of delivery, health facility, age, gravida, parity, mode of delivery, date of HIV diagnosis, sero-conversion during pregnancy [HIV diagnosis during pregnancy] (Yes/ No), gestational age at enrolment into ANC care, date of ART initiation, duration of ARV treatment (in weeks) prior to delivery, VL test done >32 weeks (Yes/ No) and the VL levels, risk status (high/low), infant status (alive/ still birth/ neonatal death), NAT result at birth and at 6 weeks, ARV regimen and duration, ART regimen and duration (if initiated) and co-trimoxazole prophylaxis. A structured data collection proforma created in EpiData Entry software was used to extract data from the registers.

### Analysis and statistics

Of the number of women who delivered, we calculated the number and proportion who were HIV-positive and who were at high/low risk for MTCT. Of the infants, we calculated number and proportion of high/low risk HIV exposed infants, number and proportion of infants who underwent NAT at birth and at 6 weeks, number and proportion of infants who received ARV prophylaxis and regimens. If the required data for high/low risk stratification were not available in the four registers or if we were unable to trace the individual patient data in the four registers, we documented it as “unknown”. We used Chi-square test to compare proportions. All analysis were done using STATA version 15.0 (STATA Corporation, College Station, Texas) statistical software.

### Ethics approval

We obtained administrative approval from the Zimbabwe Ministry of Health and Child Care to conduct this study and ethics approval from the Medical Research council of Zimbabwe and the Ethics Advisory Group of The Union, Paris, France. We got waiver from individual patient consent as this study involved review of patient records. All data were kept confidential and only the study investigators had access to it.

## Results

**Fig 1:**
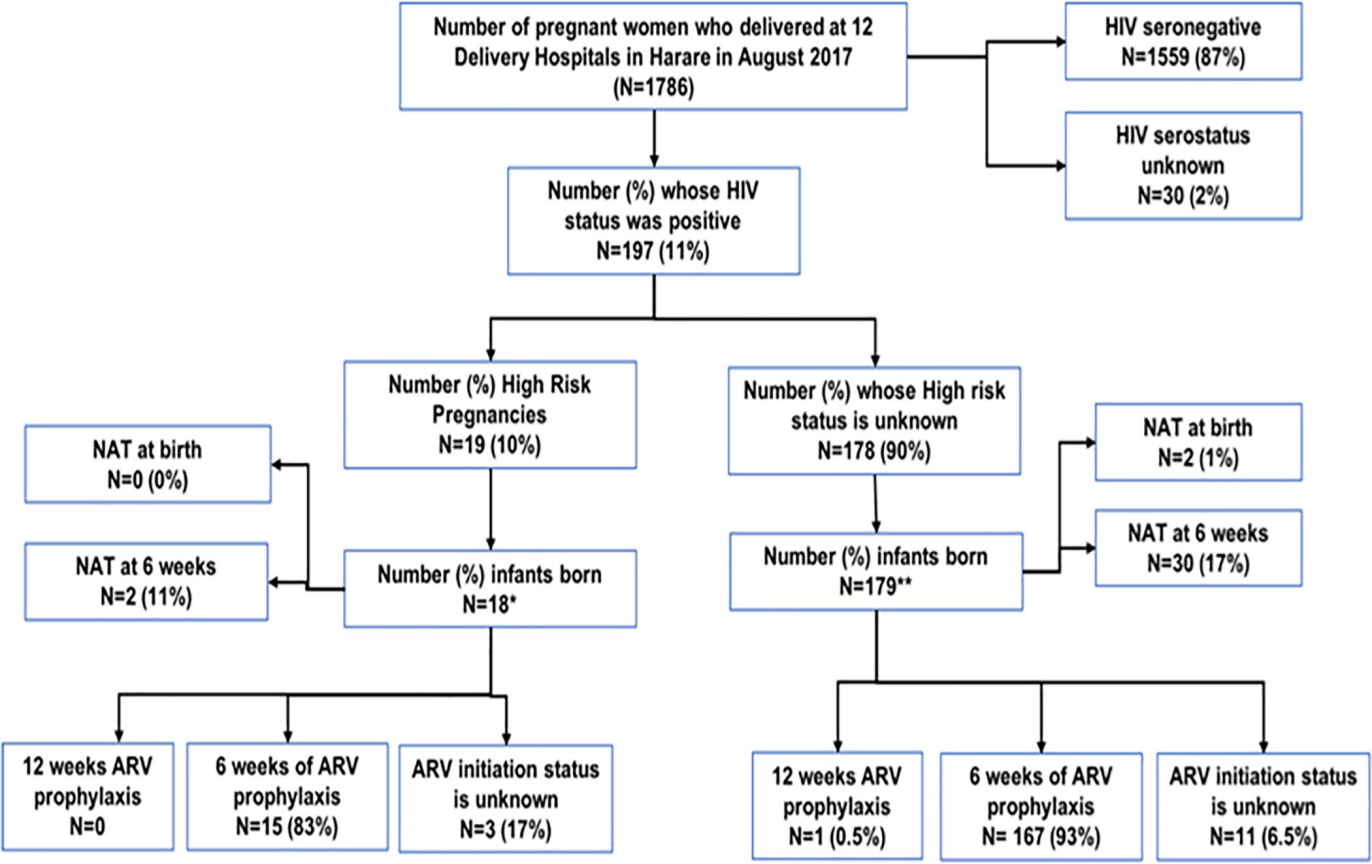
Status of implementing prevention of mother to child transmission services in 12 delivery hospitals, Harare city, August 2017*1 still birth; **1 twin; NAT = Nucleic acid test; ARV = Anti retroviral; High risk pregnancy were due to late initiation of ARV at the time of delivery.

Of the1786 pregnant women who delivered in 12 delivery clinics of Harare city in August 2017, HIV status at the time of delivery was known for 1756 (98%) of whom 197 (11%) were HIV seropositive see Fig 1. The characteristics of these women are described in Table 1.

**Table 1:** Characteristics of HIV-positive pregnant women who delivered in 12 Clinics owned by Harare City Health Department in August 2017 stratified by their risk of maternal to child transmission of HIV infection.

Variable	Total (N = 197)		High risk group (N = 19)		Unknown risk group (N = 178)		P-value
	N	%	N	%	N	%	
**Age groups (in years)**							
<19	14	7	2	11	12	7	
20–24	46	23	6	32	40	22	
25–29	51	26	6	32	45	25	0.645†
30–34	53	27	3	16	50	28	
>35 y	26	13	1	5	25	14	
Age not recorded	7	4	1	5	6	3	
*Age Median (IQR)*	28	(24–32)	25.5	(23–29)	28	(24–32)	0.0879 ‡
**Gravida**							
1	24	12	2	11	22	12	
2	74	38	9	47	65	37	
3	48	24	5	26	43	24	0.816†
4	41	21	3	16	48	27	
>4	10	5	0	0	10	6	
**Parity**							
0	1	1	0	0	1	1	
1	25	13	2	11	23	13	
2	75	38	9	47	66	37	0.960†
3	54	27	5	26	49	28	
>4	42	21	3	16	39	22	
**ART status at the time of delivery**							
On ART	176	89	0	0	176	99	
ART Naïve	19	10	19	100	0	0	NA
Unrecorded	2	1	0	0	2	1	
**Viral load done during pregnancy**							
Yes	0	0	0	0	0	0	NA
Not done	42	21	18	95	24	13	
Unknown	155	79	1	5	154	87	
**Classified as high risk by clinics**							
Yes	0	0	0	0	1	<1	NA
No	0	0	0	0	0	0	

HIV = Human immunodeficiency virus; ART = Anti-retroviral therapy; IQR = Interquartile range; NA = Not applicable; for the comparison between high risk and unknown risk group

†chi-square p-value

‡ Wilcoxon rank sum test

### Identification of “high risk” MTCT pregnancies

Of the women who were HIV positive, there were no records of viral loads, no records of whether they had sero-converted during pregnancy. However, 177 (90%) were on ART prior to delivery, 19 (10%) were newly initiated on ARV treatment at delivery, and the duration of ARV treatment during pregnancy was known only for 40 (20%) of women. Based on this information only 19 (10%) could be classified as “high risk” for MTCT and the remaining 90% lacked adequate information to classify them into high or low risk for MTCT (Fig 1). None of the clinics had recorded anyone of them as ‘high risk’ although data pointing to their "high risk" status was available, they were ART naïve at delivery. The demographic and pregnancy-related characteristics (age, gravida, parity) were similar between the pregnant women who could and could not be classified into ‘high risk’ pregnancies (Table 1).

### Nucleic acid testing for HIV exposed infants

Of the 197 women who delivered, one woman had a stillbirth and one woman gave birth to twins. Therefore, there were 197 live births (18 born to high-risk women and 179 born to women with ‘unknown’ risk). Of the 197 live births, only 2 (1%) infants had a NAT at birth (both belonging to the ‘unknown’ risk group) and 32 (16%) infants had NAT at 6 weeks (2 from the ‘high risk’ group and 30 from the ‘unknown’ risk group) (Fig 1). Of those tested by NAT at 6 weeks, one infant was initiated on ARV treatment.

### ARV prophylaxis for HIV exposed infants

Overall, 183 (93%) of 197 infants were initiated on ARV prophylaxis (167 in the ‘unknown’ risk group and 15 in the ‘high risk’ group) and the remaining 15 (7%) infants’ ARV prophylaxis status was not documented (Fig 1). Only 1 infant in the ‘unknown’ risk group received NVP + AZT prophylaxis for 12 weeks. The remaining 182 (99%) infants received 6 weeks of NVP prophylaxis.

### Cotrimoxazole prophylaxis for HIV exposed infants

Only 19 (10%) out of the 197 infants were documented to have been initiated on cotrimoxazole prophylaxis at 6 weeks. All these 19 infants belonged to the ‘unknown’ risk group.

## Discussion

This is one of the first studies reporting the status of implementing the latest national guidelines on high-risk assessment for PMTCT of HIV infection at the time of delivery and follow-up infant management during the post partum period. The HIV testing levels are high (as per the national guidelines) with 98% of the pregnant women’s HIV serostatus known at the time of delivery. Nearly all HIV seropositive pregnant women were on ART at the time of delivery. More than 90% of the HIV exposed infants were initiated on ARV prophylaxis, with almost all initiated on 6 weeks of nevirapine. However, none of the women were classified into high risk or low risk in all clinics. Due to insufficient information recorded, it was not possible for us to classify all pregnancies into high risk or low risk and assess whether the infants received correct ARV prophylaxis or not. The few high risk HEI (that we identified) did not receive 12 weeks of AZT+NVP (as per the guidelines) while one infant in the unclassified risk group got 12 weeks of AZT+NVP. The few "high risk" mothers identified by the study were missed by the clinics despite availability of adequate data (ART naïve status at delivery) at the clinics which more likely indicates poor implementation of the new 2016 PMTCT guidelines. In addition, the proportion of infants tested with NAT at birth and at 6 weeks and the proportion that were initiated on cotrimoxazole prophylaxis at 6 weeks, were negligible. This indicating serious gaps in providing appropriate clinical care, tracking, monitoring and evaluation.

The major strength of the study was that it was conducted under routine programmatic conditions with all eligible women included into the study without any exclusions and therefore the study reflects what is happening at the ground level—information that is vital for improving the situation.

The major limitation of this study was that we used a retrospective record review methodology in order to study the status of implementation of PMTCT guidelines. Hence, the reliability and validity of the study findings is dependent on the accuracy and completeness of the records/ reports. If there are deficiencies in recording, then our study findings may not reflect the ground level realities very well. The National AIDS program conducts on-site data verification visits (OSDVs) with teams visiting a sample of the health facilities (which includes delivery hospitals) every quarter to crosscheck the accuracy and completeness of records. Therefore, the deficiencies noticed in recording are evidence of a poor data management system.

The other limitation was that we collected data from the records maintained at the delivery sites. Unfortunately, results of the infant HIV test results were not available in these records and due to challenges in record linkage we were unable to identify these results from the ARV/HIV care results. As a result, we are unable to report the HIV transmission rates in this study.

The study findings have the following implications on policies and practices to improve the EMTCT services during and after delivery.

First, there is an urgent need to improve the information communication systems between the ARV clinics, ANC clinics and the delivery sites. Due to the deficiencies in this communication system, information on—the date of HIV diagnosis, whether the women have seroconverted during pregnancy, duration of ARV treatment during pregnancy, HIV viral load testing status and viral load levels—were not available at the time of delivery. This has resulted in non-identification of pregnancies as high risk of MTCT and therefore, health services have deprived infants with high-risk HIV exposure from receiving 12 weeks of dual ARV prophylaxis (NVP and AZT).

Second, nucleic acid testing of infants at birth and at 6 weeks was low according to records at the clinics. Infant HIV DNA PCR testing is done at centralized testing points with majority of specimens being tested at three major laboratories. The laboratory data management system has unique identifiers for specimens but not for HIV exposed infants. Thus it is difficult to identify individual HIV exposed infants from the data, one cannot determine whether two specimens are from two different infants or one infant as an infant`s two specimens have two different unique identifiers. Facilities are able to match results to infants using the unique specimen identity which facilities also have from the remnants of the HIV DNA PCR request form which they send to the laboratories with the specimens. HIV exposed infants who were delivered in one facility or province can have their HIV DNA PCR specimen collected from any other facility or province. However, there is no clear mechanism of tracing the infants between any two facilities particularly when testing is not done at the facility where infant was born. Compilation of the unpublished laboratory data at the national level shows that at the national level (in 2017), 49,849 HEI were identified, 55,447 dry blood samples of HEI were collected and 1561 were found to be positive. From this aggregate numbers it is not possible to know whether all HEI were identified (or not) and whether the blood samples of all eligible infants were collected on time (at birth and at 6 weeks). The data from our study shows that infants may not be identified and undergoing HIV tests in a timely manner.

Third, some of the deficiencies seen in the recording systems were due to the non-availability of checklists or fields to document the risk status of HIV seropositive pregnant women in the delivery registers. This can be improved if the delivery register formats can be revised to include the fields for recording ARV treatment number & ARV treatment site, date & site of ANC booking/registration, MTCT risk category and ARV treatment start date. The delivery register should contain reminders that prominently highlight the need to document this information and use the information for providing better services/linking the infants to appropriate care/services.

Fourth, the National AIDS program needs to start site specific analysis on the number of HIV seropositive pregnant women who deliver every month, the number and proportion who were high risk pregnancies, the number and proportion of infants who received NAT testing and ARV prophylaxis as per the guidelines so that patient-wise information on who is benefiting and who is being left out can be obtained and used to improve the services.

Lastly, the study was conducted in Harare, the capital city of Zimbabwe, which has more developed health service delivery infrastructure and human resources for health and therefore the study findings likely reflects the best case scenario when extrapolated to other parts of the country. We strongly suspect that the implementation of PMTCT guidelines in other parts of the country could be relatively poorer and therefore there is an urgent need to undertake similar assessments in other parts of the country.

The WHO 2016 guidance also include recommendations on how to implement the guidelines. World Health Organization clearly stated that guidelines should be contextualized to the implementing country. Epidemic level, country resources, comorbidities, Health system organization and capacity as well as cost effectiveness are among the factors countries need to consider when implementing the guidelines.[6] With poor viral load capacity and poor patient recording, Zimbabwe may not be in the best position to implement MTCT risk stratification basing on the requisite viral load. However, the other criteria like incident infection, absence of maternal ART or maternal ART less than 8 weeks can be used to identify "high" risk women and their babies. Risk stratification feasibility assessment also needs to be relooked with a simplified criteria based on viral load testing only, but only if viral load coverage is improved in this population of pregnant women and results also availed timely. This could be improved through the recently WHO approved Xpert HIV-1 VL machine (Cepheid, Sunnyvale, USA) for viral load point-of care testing [13] as opposed to the current centralized viral load testing, if scaled-up nationwide.

## Conclusion

Data management was poor, in particular incomplete recording of program registers and absence of clear mother-baby pair tracking and tracing mechanisms across different health delivery facilities. There were no records for viral load, sero-conversion and 90% of study participants lacked adequate information to classify them as either "high" or "low" risk MTCT. There are several gaps in implementing the PMTCT services as per the 2016 national PMTCT guidelines at the 12 delivery clinics in Harare city. In particular, there is a huge gap in identifying high risk pregnant women thus their babies are denied high risk reduction interventions like twelve weeks of dual ARV prophylaxis and priority access to HIV NAT at birth. The gaps in data management and PMTCT service provision can be addressed by urgent provision of on-going health worker training, mentorship and supportive supervision on data management and PMTCT service delivery like MTCT risk stratification, EID, infant ARV prophylaxis respectively. In the long term, introduction of an electronic health information management system which can link and share health information between facilities, laboratories, pharmacy and the Ministry of health will improve data management.
